# Identification of genes associated with persistence in *Mycobacterium smegmatis*

**DOI:** 10.3389/fmicb.2024.1302883

**Published:** 2024-02-12

**Authors:** Hemant Joshi, Divya Kandari, Subhrangsu Sundar Maitra, Rakesh Bhatnagar, Nirupama Banerjee

**Affiliations:** ^1^Laboratory of Molecular Biology and Genetic Engineering, School of Biotechnology, Jawaharlal Nehru University, New Delhi, India; ^2^Divacc Research Laboratories Pvt. Ltd., incubated under Atal Incubation Centre, Jawaharlal Nehru University, New Delhi, India

**Keywords:** persisters, *Mycobacterium smegmatis*, antibiotic tolerance, biofilm formation, *in vivo* survival

## Abstract

The prevalence of bacterial persisters is related to their phenotypic diversity and is responsible for the relapse of chronic infections. Tolerance to antibiotic therapy is the hallmark of bacterial persistence. In this study, we have screened a transposon library of *Mycobacterium smegmatis* mc^2^155 strain using antibiotic tolerance, survival in mouse macrophages, and biofilm-forming ability of the mutants. Out of 10 thousand clones screened, we selected ten mutants defective in all the three phenotypes. Six mutants showed significantly lower persister abundance under different stress conditions. Insertions in three genes belonging to the pathways of oxidative phosphorylation *msmeg_3233* (*cydA*), biotin metabolism *msmeg_3194* (*bioB*), and oxidative metabolism *msmeg_0719*, a flavoprotein monooxygenase, significantly reduced the number of live cells, suggesting their role in pathways promoting long-term survival. Another group that displayed a moderate reduction in CFU included a glycosyltransferase, *msmeg_0392*, a hydrogenase subunit, *msmeg_2263* (*hybC*), and a DNA binding protein, *msmeg_2211*. The study has revealed potential candidates likely to facilitate the long-term survival of *M. smegmatis*. The findings offer new targets to develop antibiotics against persisters. Further, investigating the corresponding genes in *M. tuberculosis* may provide valuable leads in improving the treatment of chronic and persistent tuberculosis infections.

## Introduction

*Mycobacterium tuberculosis*, the etiologic agent of tuberculosis, is reported to infect 2.6 million people in India alone (World Health Organization, 2021). Majority of the cases (90%) constitute a latent or persistent form of infection with no apparent clinical signs or symptoms (Fauvart et al., 2011; World Health Organization, 2021) and comprise the transmissible form of infectious bacteria with the ability to reactivate the disease. Tolerance and resistance are other phenotypes that are sometimes confused with the persistence (Brauner et al., 2016; Schrader et al., 2020). The latter population presents a formidable challenge in eradicating the disease (Zhang, 2014). The molecular mechanisms leading to the development of persistence are not fully understood due to several reasons. They constitute a small, heterogeneous population that is transient in nature and changes with the environment. Secondly, lack of a biological model that can mimic persistence (Zhang, 2014), and finally, due to the redundancy of mechanisms that select persisters. They constitute a subpopulation of bacteria with non-heritable characteristics that enable them to adapt to stressful conditions for long-term survival. They have the same genetic makeup as the drug-sensitive parent population, indicating that bacterial persistence is an epigenetic trait (Bigger, 1944; Keren et al., 2004; Lewis, 2010; Zhang, 2014; Torrey et al., 2016).

Persister cells are classified into three main categories: spontaneous persisters, triggered persisters, and specialized persisters (Urbaniec et al., 2022). Spontaneous persisters are slow-growing cells formed during the logarithmic phase, independent of any environmental trigger. These persisters are pre-existing in bacterial culture. The triggered persisters constitute non-growing cells produced in the stationary phase of growth due to environmental stress and exhibit an extended lag phase upon inoculation into fresh medium (Balaban et al., 2019). The specialized persisters evolve in response to specific antibiotics (Urbaniec et al., 2022). In *Escherichia coli*, tolerance to fluoroquinolones occurs by extension of the lag phase through growth-inhibiting toxin components (MazF or HipA) of the toxin-antitoxin (TA) systems (Mok and Brynildsen, 2018). Similarly, exposure of *E. coli* to ciprofloxacin reduces the proton motive force (PMF) and ATP levels in the cell, which decreases antibiotic transport, inducing drug-specific persistence (Dörr et al., 2009). In mycobacteria, variation in the levels of the catalase-peroxidase enzymes, necessary for activation of isoniazid, causes a reduction in the effective concentration of the drug, leading to drug-specific persistence (Wakamoto et al., 2013).

Biofilm formation by pathogens has emerged as one of the essential attributes contributing to the establishment of persistent infections in the host (Helaine et al., 2014). The shreds of evidence from *in vitro* experiments revealed an abundance of persisters in the biofilms; a link between the two is established, suggesting a significant role in relapses and recalcitrance of pathogenic bacteria (Keren et al., 2004; Lafleur et al., 2010; Conlon et al., 2013). The presence of persisters is believed to be responsible for antibiotic treatment failure (Van den Bergh et al., 2016; Levin-Reisman et al., 2017). It is widely accepted now that the underlying reasons behind the emergence of antibiotic-tolerant or persister cells are most likely multifactorial, especially within the host, where different interconnected metabolic processes influence each other.

Persisters have been identified in the host-dependent bacterial species, e.g., *Pseudomonas aeruginosa*, *Salmonella enterica* serovar Typhimurium, *Streptococcus suis*, *E. coli*, and *M. tuberculosis*, etc., (Helaine et al., 2014; Michiels et al., 2016). To counter host defense mechanisms, *M. tuberculosis* is reported to express various phenotypes such as biofilm growth (Chakraborty et al., 2021), tolerance to antibiotics (Ciofu et al., 2017), granuloma formation (Barry et al., 2009), survival in the host macrophage (Helaine et al., 2014), and immune evasion (Domingue et al., 2020). The hostile and restrictive environments encountered within the host macrophages and granulomatous lesions force manifestation of phenotypic heterogeneity, which enables a small population to survive/persist longer (Helaine et al., 2014; Mouton et al., 2016; Fisher et al., 2017). *In-vitro* models simulating the stress conditions experienced by mycobacteria inside the host were shown to change the metabolic state of the bacteria and generate a quiescent reservoir of bacterial cells promoting latent infection (Zhang, 2014; Lipworth et al., 2016; Joshi et al., 2021).

To identify genes regulating persistence in different bacterial species, mutagenesis is employed, which results in either decreased (e.g., *ybaL, relA, phoU, sucB, ubiF*, and *lamA*) or increased (e.g., *hipA, metG, tktA, glpD*, and *hupB*) survival propensity (Li and Zhang, 2007; Ma et al., 2010; Rego et al., 2017; Hingley-Wilson et al., 2020). These genes regulate various bacterial pathways such as TA systems, SOS response, signalling pathways, stringent response, antioxidant defence, alternative energy production, enhanced efflux or transporter activity, and phosphate metabolism, etc., (Zhang, 2014). Apparently, persisters are formed through independent parallel mechanisms, providing a significant adaptive advantage to the organism. This redundant design works in favor of the organism, as no single compound can abolish persister formation.

Latency or persistence is frequently observed in pathogenic and non-pathogenic species of mycobacteria. The fast-growing, non-pathogenic *Mycobacterium smegmatis*, which shares 70% homology with the pathogenic *M. tuberculosis*, is often used as a model to study persistence genes (Malhotra et al., 2017; Ranjitha et al., 2020). The present study is an attempt at enhancing our understanding of mycobacterial persistence by screening a transposon mutant library of *M. smegmatis* mc^2^155 strain using three key phenotypes commonly associated with persistence - antibiotic tolerance, biofilm formation, and survival in macrophages to identify genes that influence the survival of the bacteria. Our findings have revealed novel targets, which might play a role in the adaptation of *M. smegmatis* leading to persistence.

## Materials and methods

### Bacterial strains, media, growth conditions, and plasmids

The *M. smegmatis* mc^2^155 strain was used to construct and screen a transposon library. Cultures were grown in Middlebrook 7H9 liquid Broth or 7H10 Agar (Difco) solid medium enriched with 10% oleic acid-albumin-dextrose-catalase (OADC) supplement, glycerol (0.5%), and Tween-80 (0.05%). *E. coli* DH5α *λ pir* strain was used for all the cloning experiments and as the host for sequencing the transposon insertions. The chemicals used in the study were purchased from Sigma-Aldrich. A stock of antibiotics was prepared (Andrews, 2001), and dilutions were made using a 7H9 broth medium. All the strains were cultured in triplicate, for each experiment.

### Determination of minimum inhibitory concentration of antibiotics

The MIC was determined using resazurin end point microtiter assay (REMA) (Taneja and Tyagi, 2007) with some modifications. The culture was grown till the mid-exponential phase (OD ∼ 0.6−0.8) and diluted 1:100 for the assay. The initial dilutions of the antibiotics were prepared in either DMSO or sterile deionized water, and successive 2-fold dilutions were done in 100 μl of 7H9 medium supplemented with 0.05% glycerol (without Tween-80) in the microtiter plates. Antibiotic concentrations ranged from 0.005 to 4 μg/ml. In a total volume of 200 μl, each well contained 100 μl of culture containing 1 x 10^4^ CFU and 100 μl of medium containing the antibiotic. A positive control containing bacteria alone and a negative antibiotic control without culture was used to determine the bacterial viability. The plates were sealed with Parafilm and incubated at 37°C for 1−2 days. After 2 days, 30 μl of 0.02% resazurin and 12.5 μl of 20% Tween-80 were added to each well. The colour of the medium changed from blue to pink after 2 days. The fluorescence was determined using a spectrofluorimeter by excitation at 530 nm and emission at 590 nm. The lowest concentration of the antibiotic causing 90% inhibition was considered as the MIC. The percentage inhibition of viability was calculated as shown below:

Viability inhibition (%) = 1-(test well fluorescence/mean fluorescence of positive control) x 100

The MIC was tested on solid media also. A standard culture (1 x 10^4^ CFU) was plated onto antibiotic-containing (ranging from 0.005 to 4 μg/ml) and antibiotic-free control 7H10 agar plates in duplicate. The culture was spread with a sterile spreader, sealed with Parafilm, and incubated at 37°C for 3 days. The number of CFUs was recorded after 3 days. The number of live bacteria (CFU) was counted to calculate the MIC.

### Antibiotics selection for tolerant cell generation in the wild-type *M. smegmatis*

Tolerant cells generated by wild-type (WT) *M. smegmatis* were evaluated by replica plating the colonies obtained after exposure of the culture to specific antibiotics. *M. smegmatis* was treated with the antibiotics at 10X and 50X MIC (minimum inhibitory concentration), isoniazid (25 and 125 μg/ml), rifampicin (6.25 and 31.25 μg/ml), levofloxacin (0.78 and 3.90 μg/ml), and moxifloxacin (0.039 and 0.195 μg/ml) for 3 days. Samples were taken at different stages of growth, and plated on 7H10 agar medium. The colonies obtained in the previous step were replica plated with and without the drug to obtain the number of tolerant cells. The difference between the total number of colonies and the colonies able to grow in the presence of the antibiotic on sub-culturing were considered as the tolerant population for plotting a time-kill curve.

### Construction of transposon mutant library

A mutant library of *M. smegmatis* mc^2^155 was constructed by random transposon insertion mutagenesis using a φMycomarT7 phage carrying *Himar1* transposon. The transposon was transferred to *M. smegmatis* by transduction according to Lamichhane et al. (2003) with minor changes – *M. smegmatis* was grown in 7H9 containing ADC to OD_600_ of 1.2 (Lamichhane et al., 2003). The bacterial cells were centrifuged, washed two times in mycobacteriophage (MP) buffer, and the pellet was resuspended in 8 ml of the MP buffer. An aliquot was saved and used as a control for CFU counts. The cell suspension was pre-warmed to 37°C in a water bath and was mixed with approximately 2 ml of 1 x 10^11^ PFU (Plaque forming unit) or MP buffer control. The mixture was plated onto 15 cm 7H10-OADC agar plates (five to ten in number) containing 0.05% Tween-80 and 25 μg/ml kanamycin and incubated at 37°C for 2−3 days. The titer of transduction in *M. smegmatis* was ∼1.36 x 10^5^ kanamycin-resistant colonies. After harvesting the mutant colonies from the plates, they were resuspended in 7H9-OADC media containing Tween-80 (0.05%) and glycerol (15%). For storage, the cells were resuspended by sonication in water baths for two cycles of 5 s each and stored in aliquots of 1 ml at −80°C. Transposon insertion was checked by colony PCR, using forward and reverse primers (Table 1).

**TABLE 1:** Bacterial strains and plasmids were used in this study.

	Relevant characteristics	Sources
**Bacterial strains**		
*M. smegmatis* mc^2^155	*ept-1*, efficient plasmid transformation mutant of mc^2^6	ATCC
*E. coli* DH5α *λ pir*	supE44 ΔlacU169 (φ80ΔlacZM15) hsdR17recA1	DF/HCC DNA Resource CORE at Harvard Medical School
**Transposon vector**		
?MycomarT7	T7 promoter, λ pir+ OriR6K, Kanamycin resistance gene, C9 Himar1 transposon	(Sassetti and Rubin, 2003)
**Primers**		
Primers used for colony PCR		
*kan*-Fp	5′-ATGATTGAACAAGATGGATT-3′	Eurofins
*kan*-Rp	5′-TCAGAAGAACTCGTCAAGAA-3′	Eurofins
Primer used for sequencing of transposon insertion site		
*aph*-Fp	5′-CCTTCTATCGCCTTCTGTGAGT-3′	Eurofins
Primers used for amplification of transposon disrupted genes with their promoter sequence		
4044pMV261Fp	5′-ATAGGATCCGTACGTATTTGGAGG-3′	Eurofins
4044pMV261Rp	5′-ATAAAGCTTTCACGCCTGTTCACC-3′	Eurofins
2263pMV261Fp	5′-ATAGGATCCCCTACGCCCTG-3′	Eurofins
2263pMV261Rp	5′-ATAAAGCTTTCACACCATCCCGTT-3′	Eurofins
3194pMV261Fp	5′-ATAGGATCCGGCGAAATGCCAGTA-3′	Eurofins
3194pMV261Rp	5′-ATAAAGCTTTTACAGGGTGGCGT-3′	Eurofins
3455pMV261Fp	5′-ATAGGATCCGCACAGCTACCG-3′	Eurofins
3455pMV261Rp	5′-ATAAAGCTTTCAGATCTGACCGGA-3′	Eurofins
0719pMV261Fp	5′-ATAGGATCCCTGCCCCAGG-3′	Eurofins
0719pMV261Rp	5′-ATAAAGCTTTCACCGGGCGGTGA-3′	Eurofins
6655pMV261Fp	5′-ATAGGATCCTATCTGGAGCCCTT-3′	Eurofins
6655pMV261Rp	5′-ATAAAGCTTTCAGCCCCAAAC-3′	Eurofins
0392pMV261Fp	5′-ATAGGATCCGCTTCCCCTCGA-3′	Eurofins
0392pMV261Rp	5′-ATAAAGCTTTCACATCGCCGCT-3′	Eurofins
6145pMV261Fp	5′-ATAGGATCCGCCTGTCCT-3′	Eurofins
6145pMV261Rp	5′-ATAAAGCTTCTACACGCCCT-3′	Eurofins
3233pMV261Fp	5′-ATAGGATCCCTCGGCGTCTTC-3′	Eurofins
3233pMV261Rp	5′-ATAAAGCTTCTAGTAGGCGAACGAC-3′	Eurofins
2211pMV261Fp	5′-ATAGGATCCCGTGACGTCGAT-3′	Eurofins
2211pMV261Rp	5′-ATAAAGCTTTCAGCGCAGGGC-3′	Eurofins

### Selection of transposon mutants

A glycerol stock of the mutant library was inoculated in a 7H9 medium supplemented with kanamycin (25 μg/ml), grown at 37°C for 24 h, and spread on 7H10 agar plates. Based on their size, the colonies were classified as small, medium, and large. In the initial elimination stage, the segregated colonies were screened based on biofilm formation and antibiotic tolerance as follows. The colonies were grown in OADC supplemented 7H9 medium containing kanamycin in 96-well microtiter plates at 37°C for 2 days. The cultures were diluted 100 times into fresh 7H9 medium in 96-well plates. The plates were incubated at 37°C for 3 days till the OD_600_ reached the stationary phase. Levofloxacin was added to each well at a concentration of 3.9 μg/ml (50X), and incubation continued. Growth was monitored by measuring OD_600_ on days 3 and 6 and simultaneously streaked on 7H10 supplemented agar plates with no antibiotic to observe the extent of growth inhibition. The antibiotic tolerance assay was repeated with a second fluoroquinolone antibiotic, moxifloxacin. The mutants showing higher susceptibility to both levofloxacin and moxifloxacin after 3 or 6 days of exposure were selected. The antibiotic exposure was repeated to confirm the sensitivity of the mutant phenotype. Next, the selected mutants from the previous step were subjected to two cycles of biofilm formation as the second selection criteria. Those showing a defect in both criteria were subjected to the next round of tests.

### Identification of transposon insertion sites

Genomic DNA was digested using *SacII* restriction enzyme, and the fragments were self-ligated as plasmid for cloning. The competent cells of *E. coli* DH5α *λ pir* strain were transformed with the ligated mixture and spread on LB agar plates containing 50 μg/ml of kanamycin. The plates were incubated overnight at 37°C, and individual colonies were grown in 5 ml LB media with shaking at 50 μg/ml kanamycin overnight. Plasmid DNA was isolated from the cultures and sequenced with a primer designed to anneal with the 3’ end of the *aph* gene cassette in the transposon to determine the insertion site (Table 1). BLAST was used to identify the genes disrupted by insertion, with *M. smegmatis* mc^2^155 as the reference genome.

### Preparation of plasmids for genetic complementation

For complementation, plasmids were constructed by amplifying the selected genes with promoter sequence using *M. smegmatis* genomic DNA as a template with specific primers containing a 5’-BamHI restriction site and a HindIII restriction site at the 3’ end. The amplified products were digested with BamHI and HindIII restriction enzymes and ligated in pMV261 vector. The plasmids were electroporated in the respective mutant strains of *M. smegmatis*. The WT strain containing the pMV261 plasmid alone was used as a control.

### Growth determination

*M. smegmatis* and its transposon mutants were grown in triplicate in standard 7H9 liquid medium, and samples were removed at regular intervals for measuring the absorbance at 600 nm. The data presented are the mean values from three individual experiments with standard deviations.

### Influence of antibiotic time and dose on tolerance development

The strains were grown in the presence of inhibitory concentrations of levofloxacin and isoniazid and variation in the number of live/tolerant cells with time was measured. The primary cultures were grown in 10 ml of 7H9 medium containing kanamycin (25 μg/ml) at 37°C for 3 days with shaking at 150 rpm. The cultures were diluted 1:100 in 10 ml 7H9 medium and allowed to grow with shaking until their OD_600_ reached ∼ 1.8. These growth conditions were adopted for all the following experiments. An aliquot was removed to determine the CFU as a zero time point of untreated bacterial cultures. 10X MIC of levofloxacin (0.78 μg/ml) and isoniazid (25 μg/ml) were added to all the *M. smegmatis* variant strains and incubated for 6 days at 37°C with shaking. For CFU counts, aliquots were removed every 24 h and plated on 7H10 agar plates supplemented with OADC. The live cells were replica plated with and without the respective antibiotics on 7H10 agar plates and incubated at 37°C.

In another experiment, the WT *M. smegmatis*, the mutants, and their complemented strains were grown in triplicate to stationary phase, as described above. An aliquot was plated to record the CFU count of the untreated cultures. To measure the dose dependence of the strains, levofloxacin and isoniazid were added at different concentrations (10X, 20X, 30X, 40X, and 50X MIC) to all the *M. smegmatis* cultures. After incubation for 3 days at 37°C, the cultures were harvested and plated after serial dilution in 7H10 agar plates supplemented with OADC. The live cells were replica plated with and without antibiotics as described above, and the CFU counts were recorded.

### Biofilm formation assay

To examine biofilm formation, *M. smegmatis* strains were cultured in Sauton’s medium (without Tween-80) (Ojha et al., 2008). 10 μl of 3 days saturated cultures were inoculated in 1 ml of the medium and grown in 24-well, flat bottom culture plates at 37°C under stationary conditions. After 4 days of incubation, the medium and the non-adherent bacteria were removed carefully by suction, and the wells were washed three times with sterile distilled water. The attached bacteria forming the biofilm were stained for 30 min with 500 μl of 1% crystal violet (CV) at room temperature, rinsed three times with distilled water, and allowed to air dry. The stained biofilm was dissolved in 500 μl of 95% ethanol at 37°C for 30 min and used to measure optical density at 595 nm in an ELISA plate reader (TECAN, Sunrise AZreader). The assay was repeated at least three times with all the strains.

### Intracellular survival of the *M. smegmatis* variants in murine macrophages

To measure the rate of clearance of the mutants in cultured macrophages, RAW 264.7 macrophages were seeded at a density of 1 x 10^5^ cells per well in 24-well culture plates and incubated for 24 h. Mid-logarithmic phase bacterial strains were centrifuged, rinsed in PBS twice, and resuspended in DMEM containing 10% FBS medium to achieve OD_600_ ∼0.1. The bacterial suspension was kept in an ultrasonic water bath for 15 min to disaggregate the clumps and used to infect the macrophages at an MOI (multiplicity of infection) of 25:1. After 2 h, the supernatant containing floating bacterial cells was removed, and the wells were rinsed with fresh medium. Gentamicin (100 μg/ml) was added to each well and incubated for 1 h to kill bacteria attached to the surface. The infected murine macrophages were removed at different time points (0, 24, and 72 h), rinsed with PBS, and lysed in autoclaved water containing 0.1% Triton X-100. The bacterial cells in the lysate were serially diluted in PBS, followed by plating on a 7H10 agar medium enriched with OADC. The plates were incubated for 3 days at 37°C. The assay was repeated at least three times.

### Stress tolerance of *M. smegmatis* variants under *in vitro* conditions

To measure the ability of the mutant strains to tolerate stress conditions encountered in the host, the following tests were performed. Tolerance to oxidative stress was evaluated by treatment with hydrogen peroxide. The *M. smegmatis* strains were grown in supplemented 7H9 media till the stationary phase as described above. Fresh 50 ml media were inoculated with the corresponding stationary phase cultures and incubated at 37°C for 3 days. Aliquots of 5 ml were removed at 24 h intervals and treated with 10 mM hydrogen peroxide at 37°C for 2 h. An untreated culture was run in parallel as a control for comparison. At each time point, the cultures were plated and CFUs were determined.

For hypoxic stress, the *M. smegmatis* strains were grown in 10 ml 7H9 medium in glass tubes and incubated at 37°C with shaking at 150 rpm till the OD_600_ reached ∼ 0.6−0.8. The cultures were diluted 100 times in 10 ml 7H9 medium in screw-capped, flat-bottom culture tubes with 5 ml ambient air (using a headspace ratio of 0.5). Methylene blue at a concentration of 1.5 μg/ml was added to the medium before sealing to measure oxygen consumption. The culture tubes were sealed well to make them airtight and incubated with shaking at low speed at 37°C for 10 days. The depletion of oxygen was observed with the decoloration of methylene blue. Cells were harvested, plated, and the survival of the cells was determined by CFU counting.

### Growth and survival of *M. smegmatis* transposon mutants in mice

Female C57BL/6J mice were sourced from Rodent Research India Pvt. Ltd (Haryana, India). The mice were housed in sterile autoclaved micro-isolator cages maintained at temperature (26°C), light & dark cycles (12 h each), and constant humidity (30%). The animals were provided with free access to food and water during the study. The Institutional Animal Ethics Committee and Biosafety Committees of Jawaharlal Nehru University, New Delhi, India, authorized the protocols. All the experiments were performed following the animal welfare regulations of the World Organization for Animal Health. Qualified personnel performed all experiments in a biosafety level II (BSL-II) facility at the School of Biotechnology, Jawaharlal Nehru University.

To evaluate the *in vivo* survival of the WT, the transposon mutants, and their complemented strains, 6-week-old female C57BL/6J mice (three per group) were used. All the bacterial strains were grown at 37°C with shaking for 2 days, harvested, and resuspended in 0.05% PBST. The mice were injected with 1 x 10^6^ CFUs of the *M. smegmatis* strains intravenously via the tail vein. At each time point (1, 4, 7, 14, and 21 days), the mice were euthanized, and the lungs and spleen were removed aseptically and homogenized in sterile 0.05% PBST. The tissue homogenates were serially diluted in PBST and grown on 7H10 agar plates supplemented with 10% OADC and 25 μg/ml kanamycin. The plates were incubated at 37°C, and bacterial colonies were counted after 3−4 days.

## Statistical analysis

All the experiments in this study were repeated at least three times. Graphs were prepared using Graph Pad Prism 8 (GraphPad Software, La Jolla, CA). In the graphs, the data points represent the average of three independent experiments, while the error bars represent the standard deviations. Appropriate statistical tests (i.e., One-way ANOVA and Two-way ANOVA) were performed for multiple comparisons, and a *P*-value of *P < 0.05, **P < 0.01, ***P < 0.005, and ****P < 0.001 was considered statistically significant.

## Results

### Generation and screening of transposon mutants

A library of 1.36 x 10^5^ transposon mutants of the *M. smegmatis* mc^2^155 strain was generated by random insertion of *Himar1* transposon present in φMycomarT7 phage. The *Himar1* transposon requires TA dinucleotide sites in the genome for insertion. A total of 1.36 x 10^5^ random transposon mutants generated in this study suggested an approximate 19-fold targeted transposon insertions at 4.89-log_10_ TA sites (Foreman et al., 2020), in 6938 ORFs present in the genome of *M. smegmatis*. This indicated that the constructed library is likely to be saturated. From the library, 10,000 *aph-*positive clones were screened, and ∼20 were randomly checked by colony PCR for *aph* gene (data not shown). Further screening was based on the chosen phenotypes, such as antibiotic tolerance and biofilm formation. Of the total number of selected mutants, 134 showed reduced tolerance to levofloxacin and moxifloxacin, while 107 mutants were defective in biofilm formation, and ten mutants common in the above two groups, were defective in both the phenotypes. Since the probability of persistence with a defects in two of the typical features is likely to be higher, the latter were selected for detailed studies.

### Evaluation of tolerance to antibiotics in wild-type *M. smegmatis*

To generate the antibiotic sensitivity profile of WT *M. smegmatis* to select drugs for later experiments, multiple antibiotics (two first-line anti-TB drugs, isoniazid and rifampicin, and two second-line drugs, levofloxacin and moxifloxacin) were tested. After exposure of the culture to the antibiotics, response of the strain was evaluated by plating on 7H10 agar medium. Next, the colonies obtained in the previous step were replica plated to determine the number of tolerant cells. *M. smegmatis* cells that survived the initial exposure to isoniazid and rifampicin were less sensitive, as the number of live cells remained relatively high after 3 days (Supplementary Table 1). However, in the case of isoniazid, the percentage of resistant cells was relatively much smaller than the tolerant population, while treatment with rifampicin produced 80−90% of resistant cells in all the growth phases (Supplementary Table 1). Levofloxacin and moxifloxacin antibiotics were more effective in bacterial killing, but like isoniazid, they resulted in a higher proportion of tolerant than resistant cells (Supplementary Table 1). The number of surviving bacteria at 10X or 50X MICs was almost similar in all the antibiotics, suggesting that the peak antibiotic sensitivity was attained at 10X level (data not shown). Based on the above observations, isoniazid and levofloxacin at 10X MIC were selected for screening of the transposon mutants.

### Identification of genes involved in defective phenotypes

DNA sequencing of the mutants revealed the genes disrupted by transposon insertion (Table 2). The information on gene length, its location within the genome, and the putative functional product of the identified genes are shown in Table 2. Mutant strains C1, with phenotypes (antibiotic resistant, biofilm negative), C2 (antibiotic resistant, biofilm positive), and C3 (antibiotic sensitive, biofilm positive) were used as controls in the stress endurance assays. Some of the genes belonged to different pathways, such as biotin biosynthesis, cellular metabolism, glycan modifications, oxidative phosphorylation, etc. The functional aspect of the genes was derived from the KEGG (Kyoto Encyclopaedia of Genes and Genomes) database.

**TABLE 2:** Genes selected through transposon insertion of *M. smegmatis* genome.

Transposon mutants	*M. smegmatis* mc^2^155	Gene length	Gene location	Functional product	Operonic existence	*M. tuberculosis* orthologs (% identity)	Transposon insertion site
M3	*msmeg_4044*	1788 bp	4115906-4117693	GAF domain containing protein	No	Rv1429 (28%)	1538
M65	*msmeg_2263 (hybC)*	1608 bp	2346098-2347705	Hydrogenase-2, large subunit	No	−	1194
M72	*msmeg_3194 (bioB)*	1062 bp	3273453-3274514	Biotin synthase	No	Rv1589 (87%)	492
M98	*msmeg_3455 (hslR)*	384 bp	3521165-3521548	Ribosome associated heat shock protein 15	No	−	131
L20	*msmeg_0719*	1140 bp	807543-808682	Flavohemoprotein	No	Rv0385 (68%)	319
L40	*msmeg_6655*	777 bp	6709869-6710645	Hypothetical protein	No	−	214
L47	*msmeg_0392*	1515 bp	441687-443201	Putative glycosyltransferase	No	Rv1524 (58%)	686
L67	*msmeg_6145*	717 bp	6213505-6214221	Hypothetical protein	No	−	114
S12	*msmeg_3233 (cydA)*	1464 bp	3316161-3317624	Cytochrome bd ubiquinol oxidase subunit I	Yes	Rv1623c (78%)	617
S14	*msmeg_2211*	840 bp	2291531-2292370	DNA-binding protein	No	−	382
C1	*msmeg_3373*	1272 bp	3443225-3444496	Major facilitator superfamily protein MFS_1	No	−	704
C2	*msmeg_3737*	783 bp	3801921-3802703	Integral membrane protein	No	−	183
C3	*msmeg_3985*	1332 bp	4055023-4056354	Integral membrane transport protein	No	−	148

### General properties of the selected transposon mutants

To characterize the transposon mutants, the growth profile of the strains was examined, which showed an identical pattern with WT *M. smegmatis* (Supplementary Figure 1), and the MIC values of the antibiotics - isoniazid, rifampicin, levofloxacin, and moxifloxacin, were also not significantly different from the parental strain (Supplementary Table 2). These results suggest that the reduction in tolerance of the transposon mutants is not due to reduced growth rate or greater antibiotic sensitivity, but independent of the growth phase, antibiotic type, and antibiotic concentration.

### Kinetics of variation of antibiotic tolerance with time

**FIGURE 1:**
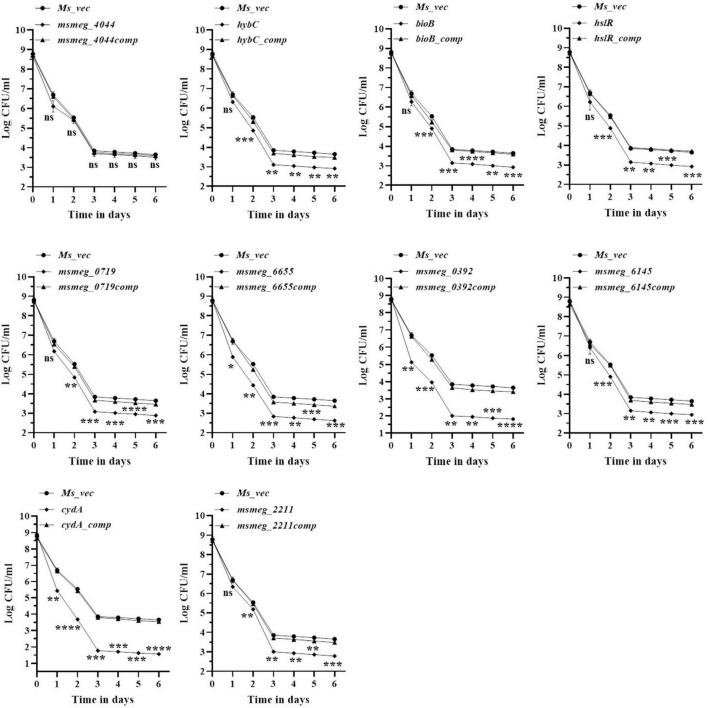
Time-dependent tolerance of *M. smegmatis* mutants against levofloxacin. Cultures were grown till OD_600_ ∼1.8, and levofloxacin was added. Samples were removed on day 1, 2, 3, 4, 5, and 6 and tolerant cell numbers were obtained by replica plating as described in Methods. The error bars represent the mean ± SD from three independent experiments. The *P*-values were calculated by two-way ANOVA with Dunnett’s multiple comparison tests. *P < 0.05, **P < 0.01, ***P < 0.005, and ****P < 0.001 in all the experiments.

**FIGURE 2:**
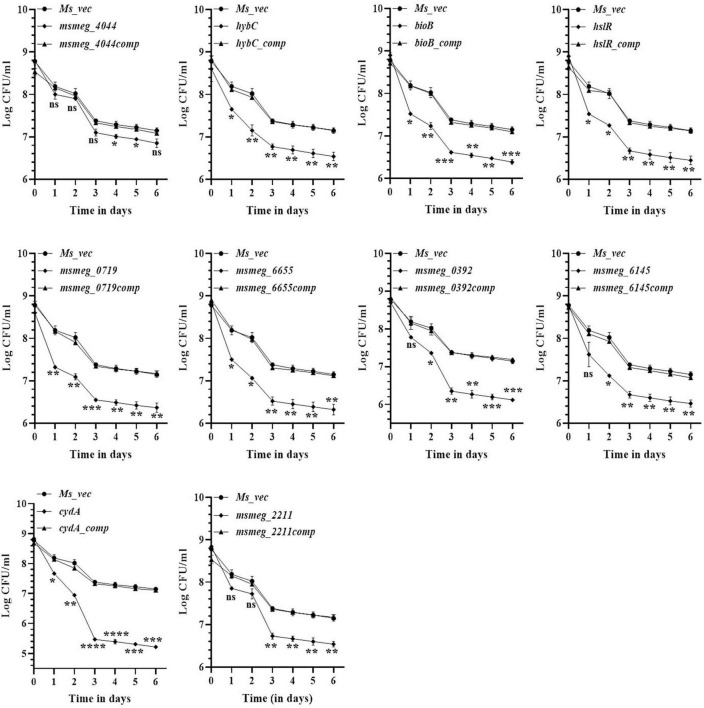
Time-dependent tolerance of *M. smegmatis* mutants against isoniazid. Cultures were grown to stationary phase (OD_600_ ∼1.8) and isoniazid was added. Samples were removed on day 1, 2, 3, 4, 5, and 6 and tolerant cell numbers were obtained by replica plating as described in Methods. The error bars represent the mean ± SD from three independent experiments. The *P*-values were calculated by two-way ANOVA with Dunnett’s multiple comparison tests. *P < 0.05, **P < 0.01, ***P < 0.005, and ****P < 0.001 in all the experiments.

To evaluate the antibiotic tolerance of the strains with time, the live cell count of the selected mutants was measured for 6 days in the presence of 10X MIC of the antibiotics. Isoniazid and levofloxacin were used followed by replica plating to exclude the resistant from the tolerant population. A time-kill curve of levofloxacin is shown in Figure 1. Out of the ten mutants, CFU counts in the *msmeg_3233* (*cydA)*, *msmeg_0392*, *msmeg_6655*, *msmeg_2211*, *msmeg_0719*, *msmeg_3194* (*bioB*), *msmeg_6145*, *msmeg_3455* (*hslR*), and *msmeg_2263* (*hybC*) strains were 2.19-log, 1.91-log, 1.02-log, 0.84-log, 0.83-log, 0.76-log, 0.76-log, 0.7-log, and 0.66-log, respectively, fold lower than the parental strain. The complemented strains showed the same growth kinetics as the WT strain. Likewise, time-kill curves at 10X MIC of isoniazid after 3 days showed a reduction in the number of live/tolerant cells of 1.97-log, 0.95-log, 0.92-log, 0.89-log, 0.83-log, 0.71-log, 0.71-log, 0.65-log, and 0.43-log, in the *cydA*, *msmeg_0392*, *msmeg_6655*, *bioB*, *hslR*, *msmeg_2211*, *msmeg_6145*, *msmeg_0719*, and *hybC* mutant strains, respectively, compared to the parental strain (Figure 2). No significant difference in the number of live cells was observed in the *msmeg_4044* mutant compared to the parental strain in response to either antibiotic. In most cases, the maximum reduction was recorded on day 3 of incubation of the strains. Taken together, the above results demonstrate that the number of tolerant cells was reduced in all the mutants. The decrease in the number of tolerant cells was significant in *cydA*, *msmeg_0392*, and *msmeg_6655* mutants relative to the WT strain and may play a role in the development of persistence in *M. smegmatis*.

### Kinetics of variation of antibiotic dose with tolerance

**FIGURE 3:**
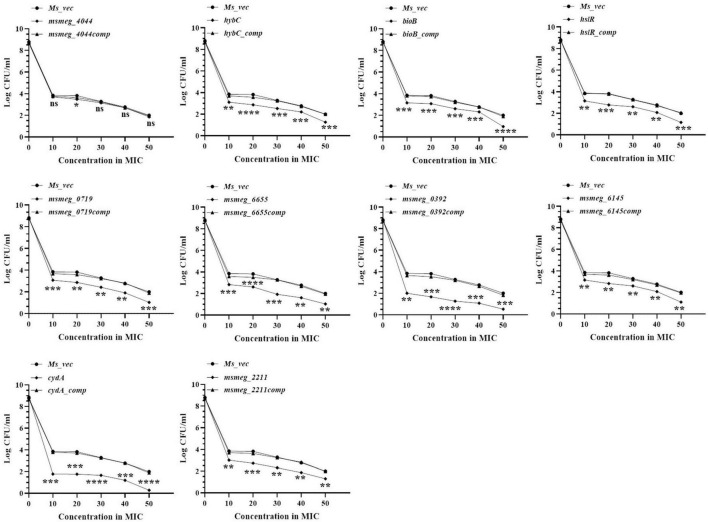
Dose-dependent tolerance of *M. smegmatis* mutants against levofloxacin. Stationary phase cultures were exposed to different concentrations of levofloxacin and incubated at 37°C for 3 days. Samples were removed on day 3 and tolerant cell numbers were obtained by replica plating as described in Methods. The error bars represent the mean ± SD from three independent experiments. The *P*-values were calculated by two-way ANOVA with Dunnett’s multiple comparison tests. *P < 0.05, **P < 0.01, ***P < 0.005, and ****P < 0.001 in all the experiments.

**FIGURE 4:**
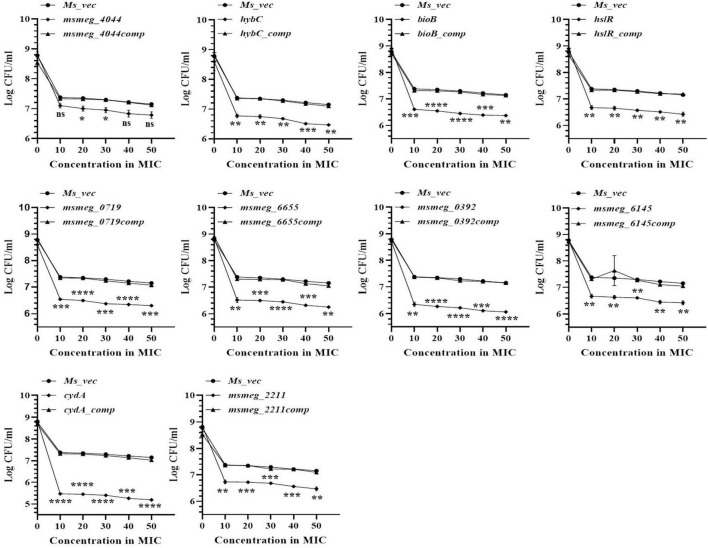
Dose-dependent tolerance of *M. smegmatis* mutants against isoniazid. Stationary phase cultures were exposed to different concentrations of isoniazid and incubated at 37°C for 3 days. Samples were removed on day 3 and tolerant cell numbers were obtained by replica plating as described in Methods. The error bars represent the mean ± SD from three independent experiments. The *P*-values were calculated by two-way ANOVA with Dunnett’s multiple comparison tests. *P < 0.05, **P < 0.01, ***P < 0.005, and ****P < 0.001 in all the experiments.

The number of tolerant cell counts at varying antibiotic concentrations (10X−50X) was measured after 3 days of exposure. The change in the number of live cells with increasing concentrations of levofloxacin is shown in Figure 3. Maximum antibiotic susceptibility with respect to the WT was observed between 10X and 20X MIC in most of the mutants. The fold decrease in the live cell counts of *cydA*, *msmeg_0392*, and *msmeg_6655* mutants was 2.17-log, 2.21-log, and 1.21-log, respectively, relative to the parent strain, over the entire range of antibiotic concentrations (Figure 3). The mutant strains complemented with the respective WT genes showed full recovery in the number of surviving cells. The reduction in the levels of live cells in the remaining transposon mutant ranged between 0.5-log to 1-log compared to the WT and complemented strains (Figure 3). At higher levofloxacin concentrations, the trend in the sensitivity of the strains followed the same pattern as before. In the case of isoniazid, the maximum reduction in the counts of drug-tolerant cells occurred at 10X MIC in all the mutants, as shown in Figure 4. The *cydA* mutant recorded a maximum reduction of ∼ 2-log, while in the remaining mutants, the decrease in the number of tolerant cell counts varied between 0.5-log and 1-log. Further, similar results were obtained at higher concentrations of isoniazid (Figure 4). The sensitivity of *msmeg_4044* mutant strain to both levofloxacin and isoniazid was not significantly different from the WT strain.

### Effect on Biofilm formation by *M. smegmatis* strains

**FIGURE 5:**
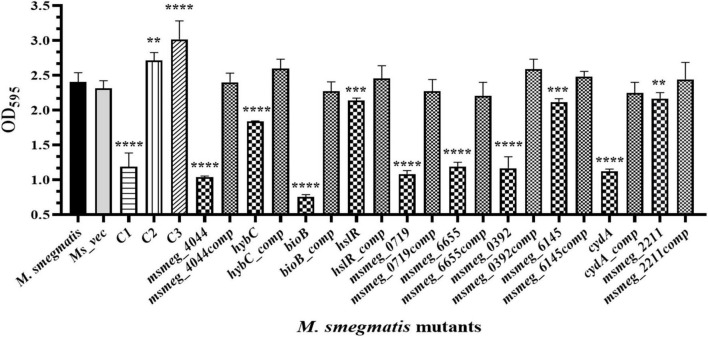
Biofilm formation by transposon mutants. Quantitative representation of the biofilm formed by the mutants. The stationary phase saturated culture was inoculated in Sauton’s medium as above and incubated at 37°C for 4 days. The medium was removed carefully, and adherent biofilms were stained with crystal violet for optical density measurement at 595 nm. The error bars indicate mean ± SD from three independent experiments. *P < 0.05, **P < 0.01, ***P < 0.005, and ****P < 0.001 in all the experiments.

The ability to form biofilm is a hallmark of persistent pathogens causing chronic infections. Hence, it was used as a marker to measure tolerance of *M. smegmatis*. Biofilm development on hydrophobic, plastic surface by the mutants is shown in Figure 5. The CV staining confirmed variable degrees of defect in biofilm formation by all the mutants relative to *M. smegmatis* or the complemented strains. The *msmeg_4044*, *bioB*, *msmeg_0719*, *msmeg_6655*, and *msmeg_0392* variants showed a significant reduction in CV binding, while *hybC*, *hslR*, and *msmeg_2211* mutant strains were affected to a lesser degree. The control mutant stains C1 showed ∼50% reduction, while C2 and C3 showed no defect in biofilm development, as expected (Figure 5). Our results suggest that the disrupted genes in the selected transposon mutants are directly or indirectly involved in the process of biofilm formation in *M. smegmatis*, eventually helping the cell to survive.

### Survival of the mutants in mouse macrophages

**FIGURE 6:**
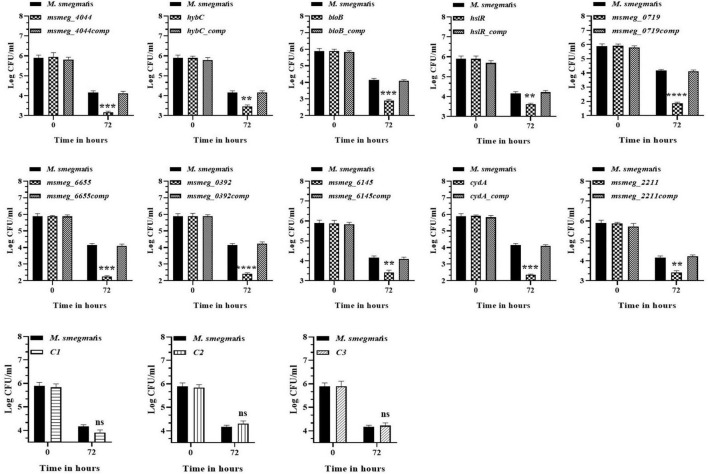
Survival of *M. smegmatis* mutants in murine macrophages. Cultured RAW 264.7 cells were infected with *M. smegmatis* strains at m.o.i of 25:1. The cells were harvested at 0, 24, and 72 h post-infection and CFUs counts were obtained by plating appropriate dilutions on 7H10 agar plates supplemented with kanamycin (25 μg/ml). The error bars represent mean ± SD from three individual experiments. The *P*-values were calculated by two-way ANOVA with Dunnett’s multiple comparison tests. *P < 0.05, **P < 0.01, ***P < 0.005, and ****P < 0.001 in all the experiments.

To evaluate the ability of the mutants to tolerate the hostile conditions *in vivo*, they were infected in the murine macrophage cell line. The clearance rate of all the mutant strains from the RAW 264.7 macrophages was faster than the WT strain from 24 h onwards. After 72 h, *msmeg_0719* recorded more than 2-log; *msmeg_6655*, *cydA*, *msmeg_4044*, *bioB*, and *msmeg_0392* between 1-log to 2-log; and *msmeg_6145*, *hybC*, *hslR*, and *msmeg_2211* mutants recorded about 0.5-log, lower counts than the WT and the complemented strains (Figure 6). Overall, all ten transposon mutants displayed a variable degree of defect in intracellular survival within the murine macrophages. The clearance time of the control strains C1, C2, and C3 were similar to the WT strain, suggesting that a defect in single phenotype is not enough to cause a significant reduction in the survival of *M. smegmatis*.

### Stress response of *M. smegmatis* variants

**FIGURE 7:**
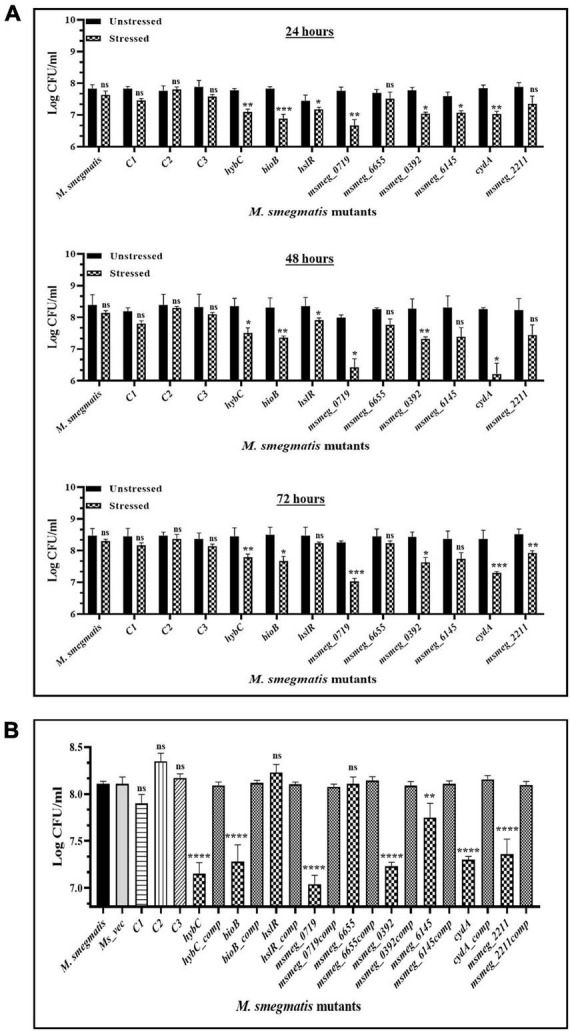
Survival of *M. smegmatis* mutants under *in vitro* stress conditions. **(A)** Stationary phase cells of bacterial strains were washed and exposed to 10 mM hydrogen peroxide at 37°C for 2 h at different time intervals. **(B)** All the bacterial strains were incubated in hypoxic conditions for 10 days at 37°C. The CFUs were recorded by plating on 7H10 agar plates supplemented with kanamycin. The error bars represent the mean ± SD from three independent experiments. The *P*-values were calculated by two-way ANOVA with Dunnett’s multiple comparison tests. *P < 0.05, **P < 0.01, ***P < 0.005, and ****P < 0.001 in all the experiments.

To examine the stress response of the mutants, the latter were exposed to conditions encountered by mycobacteria during infection, e.g., oxidative stress and hypoxic environment, which reduced the number of survivors in all the mutants compared to the WT strain. All the strains displayed greater sensitivity to oxidative stress at the peak of the growth phase after 48 h. The *msmeg_0719* and *cydA* mutants appear more sensitive than others in the group, showing maximum reduction of ∼ 2-log in CFU compared to the WT strain (Figure 7A). While ∼ 1 log-fold reduction was observed in the *hybC*, *bioB*, *msmeg_0392*, and *msmeg_6145* mutants over the WT strain (Figure 7A). Of the nine transposon mutants tested, *msmeg_4044*, *hslR*, and *msmeg_6655* mutants, were not affected significantly during exposure to oxidative stress. The number of live cells in the control mutant strains was similar to WT strain, reiterating the synergistic action of multiple genetic loci in adaptation and long-term survival of *M. smegmatis* (Figure 7A).

Some of the mutants showed higher sensitivity to hypoxic stress (Figure 7B). The strain *msmeg_0719* showed maximum reduction of ∼1-log in CFU counts, while *hybC*, *bioB*, *msmeg_0392*, *cydA*, and *msmeg_2211* were all affected adversely with lower than 1-log-fold change under hypoxic conditions. As in oxidative conditions, the *hslR* and *msmeg_6655* mutants, were not affected significantly (Figure 7B).

### Survival of the transposon mutants in mice

**FIGURE 8:**
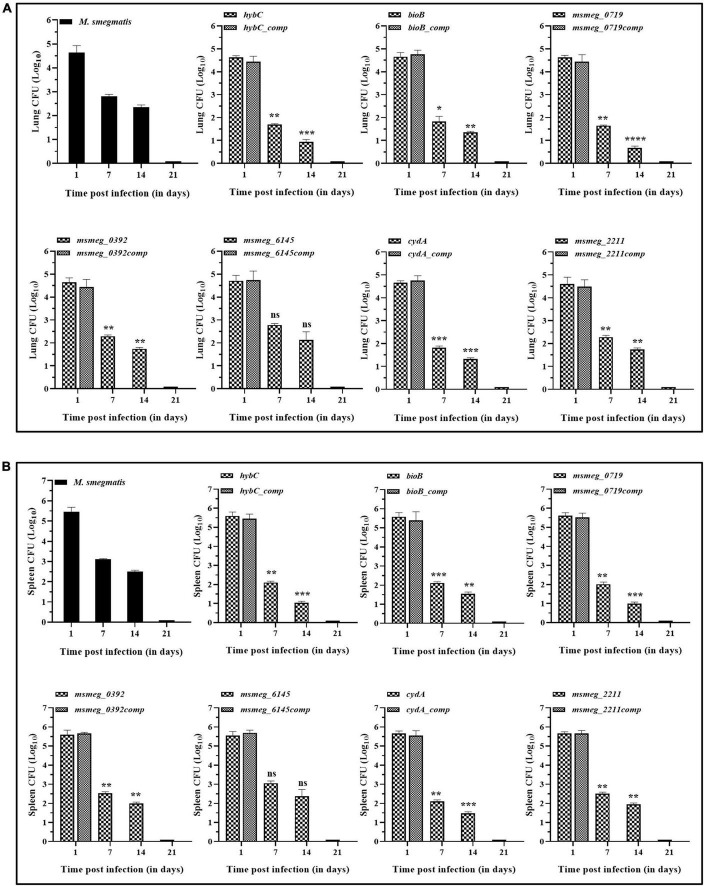
Persistence of *M. smegmatis* mutants in lungs and spleen of mice. C57BL/6J mice were injected intravenously with 1 x 10^6^ CFUs of actively growing cells. Survival of the bacteria in the **(A)** lungs and **(B)** spleen was determined on day 1, 7, 14, and 21 by plating appropriate dilutions on 7H10 agar plates containing kanamycin (25 μg/ml). At day 21 complete bacterial clearance was observed. The data for complemented strain was plotted for day 1, as it indicates the gene function was restored. The statistical significance was determined by comparing mutant strains with the wild type at different time points of infection. The error bars represent the mean ± SD from three independent experiments. The *P*-values were calculated by two-way ANOVA with Dunnett’s multiple comparison tests. *P < 0.05, **P < 0.01, ***P < 0.005, and ****P < 0.001 in all the experiments.

The degree of tolerance of the strains *in vivo* was tested in mice. C57BL/6J mice were injected with *M. smegmatis* strains, and CFU in the lungs and spleen were enumerated. In general, the bacterial burden was substantially reduced in the organs by 7 days and was fully cleared at 21 days of infection. After 7 days of infection, the bacterial counts in the lungs and the spleen of several mutants, namely *msmeg_0719*, *hybC*, *bioB*, and *cydA*, were ∼1-log lower than the WT and the complemented strains. The CFU counts decreased further at 14 days in the lungs of *msmeg_0719*, *msmeg_0392*, *hybC*, *bioB*, *cydA*, and *msmeg_2211* mutants (ranging between 1.7-log to 0.66-log) with respect to the parent strain, while *msmeg_6145* was not significantly different from the *M. smegmatis* control (Figure 8A). Likewise, the number of surviving cells in the spleens of *msmeg_0719*, *msmeg_0392*, *hybC*, *bioB*, *cydA*, and *msmeg_2211*, mutants was 1.52-log, 1.48-log, 1.04-log, 0.97-log, 0.6-log, and 0.53-log lower than the WT and complemented strains after 14 days (Figure 8B). Overall, initially (7 days) the rate of clearance of WT bacteria in the spleen was higher (2.35-log) than in the lungs (1.82-log), but at later time point (14 days), the clearance rate was nearly same in both the organs. These results indicate that due to the disruption of some genes, the six mutants mentioned above displayed a significantly lower propensity to survive under *in vivo* conditions than the parental strain, and the genes affected in the latter may play a role in bacterial persistence in the host tissues.

## Discussion

It has become abundantly clear that there is no single mechanism to explain the phenomenon of persistence in bacteria (Hu and Coates, 2005; Zhang, 2014; Urbaniec et al., 2022). The ability of *M. tuberculosis* and *M. smegmatis* to form persister cells *in vitro* and *in vivo* has been demonstrated in several studies (Grant et al., 2012; Manina et al., 2015; Torrey et al., 2016). Here, we have adopted three different screening criteria intimately linked to bacterial persistence, e.g., antibiotic tolerance, biofilm formation, and survival *in vivo* in the macrophages, to screen a transposon mutant library of *M. smegmatis* mc^2^155. The rationale for using multiple phenotypes for selection was based on the assumption that a gene may control multiple phenotypes indirectly by participating in different metabolic pathways. Hence, disruption of a genetic locus, manifesting defects in multiple phenotypes is likely to be involved in persistence development in a strain. Also, supported by our observations that multiple disabilities in a strain increased the susceptibility of the organism to host clearance mechanisms.

The *msmeg_*3233 gene encodes the CydA subunit of cytochrome bd oxidase. In mycobacteria, the electron transport chain (ETC) terminates through the actions of two-terminal oxidases: an aa3-type cytochrome c oxidase and cytochrome bd oxidase. The latter is involved in the generation of the PMF to fuel energetic processes in the cell membrane (Cook et al., 2014). The cytochrome bd oxidase has been implicated in the adaptation of *M. tuberculosis* to host immunity-induced stress microenvironments during infection (Giuffrè et al., 2012; Forte et al., 2016). In *M. smegmatis*, although not essential under normal oxygen conditions, maintaining mycobacterial respiration is indispensable during hypoxic conditions (Aung et al., 2014; Jeong et al., 2018). In different mycobacterial species, *cydA* expression was induced by cell wall inhibitors, e.g., Q203, bedaquiline, nitroimidazoles, augmentin, potassium cyanide, and clofazimine, emphasizing its importance in stress alleviation and survival (Koul et al., 2014; Boot et al., 2017; Kalia et al., 2017). Based on these reports, cytochrome bd oxidase appears to relieve the pressure on the ETC and allow the bacteria to persist longer by maintaining membrane potential in the absence of cytochrome bcc1-aa3 complex and ATP synthase activities. In the light of previous studies, the defects displayed by *cydA* mutant in all the parameters tested, make a strong case for a key role in global stress management and survival of *M. smegmatis*.

Disruption of the *msmeg_0719* gene is attributed to faster clearance of the mutant, making it sensitive to intracellular stress conditions; the most important among them were oxidative stress, hypoxia, and survival in the mouse macrophages. Oxidative stress is one of the major causes of death of intracellular pathogens. The reactive oxygen species released within macrophages cause lipid peroxidation of the lipids-rich cell walls of mycobacteria and produce toxic methylglyoxal and D-lactate (Rachman et al., 2006). In *M. tuberculosis*, the corresponding ortholog Rv0385 encodes a peripheral membrane-associated flavohemoglobin, which catalyzes D-lactate to pyruvate and protects the respiratory membranes from toxic ROS-mediated killing (Gupta et al., 2012). Secondly, under hypoxic conditions, the heme domain of the protein transfers the electrons acquired from D-lactate to the electron transport chain in the cell membrane to produce energy at a low level, keeping the bacteria viable in a non-dividing state within the host (Gupta et al., 2012). The precise mechanism of Rv0385 in the persistence of *M. tuberculosis* would require further investigation. Since it has been conserved in the genome of a number of the pathogenic and non-pathogenic mycobacterial strains (Gupta et al., 2011), it could be a potential target for combating long-term, non-replicative survival of mycobacteria.

In biological systems, biotin acts as a cofactor for enzymes that catalyze metabolic reactions e.g., membrane lipid synthesis, tricarboxylic acid cycle, and amino acid metabolism etc., (Salaemae et al., 2016). Biotin is synthesized from pimeloyl-CoA using four enzymes encoded by *bio*F, *bioA*, *bioD*, and *bioB* genes organized in an operon in *M. tuberculosis* and in two separate ones in *M. smegmatis* (Tang et al., 2014; Salaemae et al., 2016). *BioB* gene plays a key role in biotin synthesis in both the mycobacterial species (Fan et al., 2015; Lazar et al., 2017). Development of auxotrophy in the *bio*B gene mutant (Supplementary Figure 2) and significant reduction in the live cell counts under different stress conditions, e.g., bactericidal antibiotics, oxidative stress, intracellular survival, and hypoxia, reiterate the importance of biotin synthesis in growth and global stress management in *M. smegmatis*.

The reduction in biofilm growth of the *bioB* mutant is most likely a direct effect of disruption in biotin synthesis by the strain. Further, in a *bioB* mutant of *M. bovis*, increased susceptibility to Para-amino salicylic acid and rifampicin in biotin-limiting conditions was attributed to lack of biotin, implying its significance in antibiotics tolerance in mycobacteria (Howe et al., 2018). Thus, mutation in the *bioB* gene appears to be responsible for the defects in both the phenotypes selected as our screening criteria in *M. smegmatis.* Biotin is known to play a critical role in the biofilm growth of bacteria, which protects them from the hostile conditions in the host (Chakraborty et al., 2021). Ojha and Hatfull (2007) have reported activation of *bioB* gene transcription in the late stages of biofilm formation in *M. smegmatis*. Considering the degree of attenuation of the mutant, a potential role of the *bioB* gene in the slow-growing, non-replicating, persistent state of *M. tuberculosis* cannot be ignored.

The *msmeg_0392* gene encodes a putative glycosyltransferase enzyme involved in carbohydrate transport and metabolism (Vera-Cabrera et al., 2007). It belongs to the Gtf3 family of proteins, necessary for synthesizing glycosylated glycopeptidolipids (GPLs) in *M. smegmatis* (Deshayes et al., 2005). It has been reported that *M. smegmatis* produces triglycosylated GPLs during the late stationary phase under low carbon conditions (Ojha et al., 2002), implicating their role in shielding the cells in unfavorable conditions. Thus, the enhanced susceptibility of the *msmeg_0392* gene mutant to multiple stress conditions merits further investigations to learn about its mechanism of action in the context of persistence. Additionally, the study of *M. tuberculosis* ortholog (*rv1524*) may also reveal necessary attributes for the long-term survival of the pathogen.

The *msmeg_2263* gene is involved in molecular hydrogen (H_2_) metabolism in mycobacteria (Berney et al., 2014). The *msmeg_2263* (*hybC*) and *hybA* genes encode the large and small subunits, respectively, of the nickel and iron-dependent hydrogenase-2 enzyme in *M. smegmatis.* The hydrogenases catalyze the conversion of molecular hydrogen into electrons and protons. The protons are responsible for generating the PMF, whereas the electrons enter aerobic or anaerobic respiratory chains (Benoit et al., 2020). The hydrogenases maintain the flow of reducing equivalents to the ETC during conditions of energy limitation. Genetic studies have shown that the *msmeg_2263* gene is induced during starvation and hypoxia, which promotes survival by oxidizing H_2_ in *M. smegmatis* (Berney and Cook, 2010; Cook et al., 2014). Further, under hypoxia, mycobacteria switch on hydrogenases to adapt and, like anaerobic bacteria, transfer electrons in the absence of exogenous electron acceptors through the unique electron carriers—ferredoxins (Berney and Cook, 2010). Based on the available information, it is clear that the role of hydrogenases in facilitating long-term survival under low carbon/energy and oxygen conditions is crucial for *M. smegmatis*, making it a good target for preventing persister formation. Furthermore, it would be interesting to identify counterparts of the gene in *M. tuberculosis* and study their role in persistence.

The emerging scenario from this study highlights the cooperative role of (i) alternate routes of energy generation in low carbon and hypoxic situations and (ii) protection from free radicals generated by oxidative burst in the phagocytic cells, as the key factors that enable mycobacteria to outlive the hostile environment in the host. Presently we are unable to propose a mechanism of action of other genes that were picked up by our screen e.g., *hslR*, a ribosome-associated heat shock protein15; *msmeg_6655*, and *msmeg_6145*, hypothetical proteins; *msmeg_2211*, a DNA-binding protein; and *msmeg_4044*, a GAF domain-containing protein in mycobacteria, due to lack of comprehensive information about their functional nature and will be the subject of our future studies.

In comparison to the pathogenic mycobacterial strains, the faster growth rate of *M. smegmatis* may raise questions about its suitability as a model to study tolerance/ persistence. However, the long-term survival of an organism requires multiple attributes other than growth rate to tolerate the hostile host environment. The literature is replete with studies performed in *M. smegmatis*, which provide valuable insight into the unique characteristics of the genus Mycobacterium (Sharbati-Tehrani et al., 2005; Malhotra et al., 2017; Ranjitha et al., 2020; Sparks et al., 2023). Using *M. smegmatis* as a model has the advantage of acquiring a large volume of data in a short time for studying the pathogenic traits of a species. This study was aimed to do the groundwork for identifying potential genes (with orthologs in *M. tuberculosis*) involved in the long-term survival of mycobacteria. We assumed that investigating the conserved stress-inducible housekeeping genes of *M. smegmatis* may reveal information applicable to *M. tuberculosis* persistence.

It is challenging to identify genes involved in persistence development due to the transient nature of the phenotypes and the redundancy of mechanisms promoting long-term survival. Each effort reveals new targets, providing insight to understand the process. Likewise, this study has identified some target genes in *M. smegmatis*. To our knowledge, the five genes have not been described in the context of the persistence of mycobacteria. These genes participate in vital metabolic pathways, including alternate methods of energy production, hydrogen metabolism, biotin synthesis, biofilm formation, and carbohydrate metabolism. Some of these genes have orthologs in *M. tuberculosis* and offer possibilities for developing novel antibiotics to treat chronic and persistent tuberculosis infections.

## Data availability statement

The original contributions presented in this study are included in the article/Supplementary material, further inquiries can be directed to the corresponding authors.

## Ethics statement

The animal study was approved by the Institutional Animal Ethics Committee of Jawaharlal Nehru University. The study was conducted in accordance with the local legislation and institutional requirements.

## Author contributions

HJ: Conceptualization, Data curation, Formal Analysis, Investigation, Methodology, Validation, Writing – original draft, Writing – review & editing. DK: Conceptualization, Formal Analysis, Methodology, Writing – review & editing. SM: Supervision, Visualization, Writing – review & editing. RB: Funding acquisition, Resources, Supervision, Visualization, Writing – review & editing. NB: Conceptualization, Data curation, Formal Analysis, Investigation, Methodology, Supervision, Writing – review & editing.
