# Supplementary material for: Identification of genes associated with persistence in Mycobacterium smegmatis
Source: Front Microbiol. 2024 Feb 12;15:1302883. doi: 10.3389/fmicb.2024.1302883 (PMC10894938; doi:10.3389/fmicb.2024.1302883)
Supplement: Supplementary file 1 [file Data_Sheet_1.PDF]

*Supplementary Material*

# Identification of Genes Associated with Persistence in *Mycobacterium smegmatis*

**Hemant Joshi[1], Divya Kandari[1,2], Subhrangsu Sundar Maitra[1], Rakesh Bhatnagar[1*], Nirupama Banerjee[1,2*]**

*** Correspondence: Nirupama Banerjee**: nirupamaban@yahoo.com

*** Co-correspondence: Rakesh Bhatnagar**: rakeshbhatnagar@jnu.ac.in

**Result:**

**Table S1**. Evaluation of antibiotic-resistant and sensitive cells in the wild-type *M. smegmatis* mc$^2$155 strain. Different growth phase cultures (lag, log, and stationary phase) were exposed to a 50X MIC dose of the four antibiotics (isoniazid, rifampicin, levofloxacin, and moxifloxacin) for 24 h, 48 h, and 72 h. The cultures containing a mixture of antibiotic-resistant and tolerant cells were washed and plated on 7H10 agar medium at each time point. Next, the colonies obtained in the previous step were replica plated in the presence and absence of respective antibiotics to determine the frequency of antibiotic-resistant and tolerant cells. The data were obtained from three independent experiments and mean ± SD values were mentioned in the table.

| Growth phase | Incubation time | Resistant cells (in %) | Tolerant cells (in %) | Growth phase | Incubation time | Resistant cells (in %) | Tolerant cells (in %) |
|---|---|---|---|---|---|---|---|
| **Isoniazid** (125 µg/ml) | | | | **Rifampicin** (31.25 µg/ml) | | | |
| **Lag phase** | 24 hours | 4 ± 0.02 | 96 ± 0.15 | **Lag phase** | 24 hours | 98 ± 0.08 | 2 ± 0.04 |
| | 48 hours | 3 ± 0.05 | 97 ± 0.08 | | 48 hours | 97 ± 0.11 | 3 ± 0.02 |
| | 72 hours | 2 ± 0.01 | 98 ± 0.05 | | 72 hours | 95 ± 0.18 | 5 ± 0.10 |
| **Log phase** | 24 hours | 32 ± 0.07 | 68 ± 0.09 | **Log phase** | 24 hours | 96 ± 0.21 | 4 ± 0.02 |
| | 48 hours | 50 ± 0.11 | 50 ± 0.11 | | 48 hours | 91 ± 0.09 | 9 ± 0.08 |
| | 72 hours | 30 ± 0.10 | 70 ± 0.18 | | 72 hours | 88 ± 0.11 | 12 ± 0.05 |
| **Stationary phase** | 24 hours | 30 ± 0.15 | 70 ± 0.09 | **Stationary phase** | 24 hours | 95 ± 0.08 | 5 ± 0.07 |
| | 48 hours | 45 ± 0.12 | 55 ± 0.07 | | 48 hours | 90 ± 0.15 | 10 ± 0.12 |
| | 72 hours | 15 ± 0.08 | 85 ± 0.15 | | 72 hours | 83 ± 0.15 | 17 ± 0.05 |
| **Levofloxacin** (3.90 µg/ml) | | | | **Moxifloxacin** (0.195 µg/ml) | | | |
| **Log phase** | 24 hours | 1 ± 0.03 | 99 ± 0.17 | **Log phase** | 24 hours | 1 ± 0.05 | 99 ± 0.20 |
| | 48 hours | 4 ± 0.05 | 96 ± 0.11 | | 48 hours | 9 ± 0.04 | 91 ± 0.11 |
| | 72 hours | 1 ± 0.02 | 99 ± 0.08 | | 72 hours | 7 ± 0.08 | 93 ± 0.15 |
| **Stationary phase** | 24 hours | 2 ± 0.01 | 98 ± 0.15 | **Stationary phase** | 24 hours | 5 ± 0.03 | 95 ± 0.12 |
| | 48 hours | 4 ± 0.03 | 96 ± 0.05 | | 48 hours | 10 ± 0.07 | 90 ± 0.05 |
| | 72 hours | 1 ± 0.03 | 99 ± 0.08 | | 72 hours | 10 ± 0.11 | 90 ± 0.09 |

**Table S2**. Minimum inhibitory concentrations (MIC) of different antibiotics against *M. smegmatis* and its transposon mutants. The MIC of four different antibiotics (i.e., isoniazid, rifampicin, levofloxacin, and moxifloxacin) was quantified against *M. smegmatis* variants using two different methods, i.e., REMA and CFU assay. The data were obtained from three independent experiments, and similar results were obtained each time.

| Transposon mutants | Minimum inhibitory concentrations (in µg/ml) | | | | | | | |
|---|---|---|---|---|---|---|---|---|
| | Isoniazid | | Rifampicin | | Levofloxacin | | Streptomycin | |
| | REMA | CFU assay | REMA | CFU assay | REMA | CFU assay | REMA | CFU assay |
| ***M. smegmatis*** | 1.25 | 2.5 | 0.625 | 0.625 | 0.078 | 0.078 | 0.25 | 0.25 |
| ***msmeg_4044*** | 1.25 | 2.5 | 0.625 | 0.625 | 0.078 | 0.078 | 0.25 | 0.25 |
| ***hybC*** | 1.25 | 2.5 | 0.625 | 0.625 | 0.078 | 0.078 | 0.25 | 0.25 |
| ***bioB*** | 1.25 | 2.5 | 0.625 | 0.625 | 0.078 | 0.078 | 0.25 | 0.25 |
| ***hslR*** | 1.25 | 2.5 | 0.625 | 0.625 | 0.078 | 0.078 | 0.25 | 0.25 |
| ***msmeg_0719*** | 1.25 | 2.5 | 0.625 | 0.625 | 0.078 | 0.078 | 0.25 | 0.25 |
| ***msmeg_6655*** | 1.25 | 2.5 | 0.625 | 0.625 | 0.078 | 0.078 | 0.25 | 0.25 |
| ***msmeg_0392*** | 1.25 | 2.5 | 0.625 | 0.625 | 0.078 | 0.078 | 0.25 | 0.25 |
| ***msmeg_6145*** | 1.25 | 2.5 | 0.625 | 0.625 | 0.078 | 0.078 | 0.25 | 0.25 |
| ***cydA*** | 1.25 | 2.5 | 0.625 | 0.625 | 0.078 | 0.078 | 0.25 | 0.25 |
| ***msmeg_2211*** | 1.25 | 2.5 | 0.625 | 0.625 | 0.078 | 0.078 | 0.25 | 0.25 |

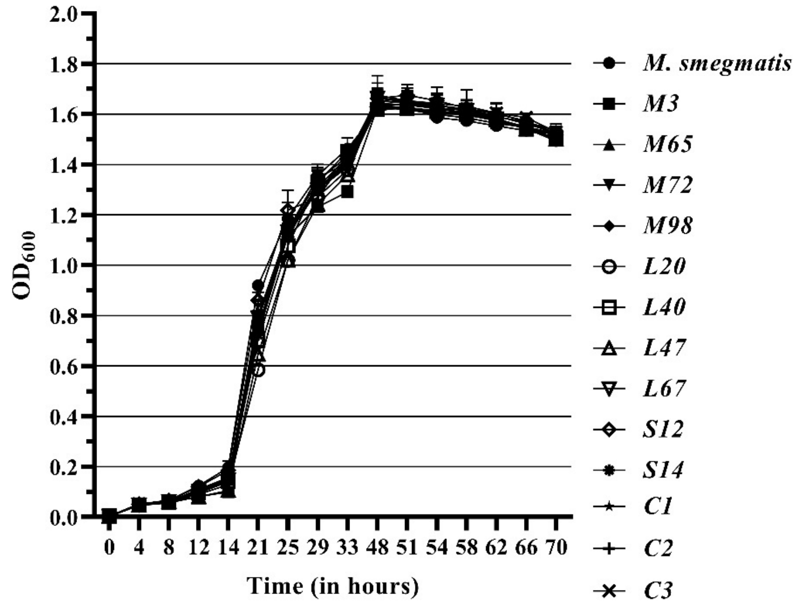


**Figure S1**. Growth curves of *M. smegmatis* and its transposon mutants. The *M. smegmatis* variants were grown in 7H9 medium with shaking, and samples were taken at different time points to measure optical density at 600 nm. The data were obtained from three independent experiments, and error bars represent the mean $\pm$ SD.

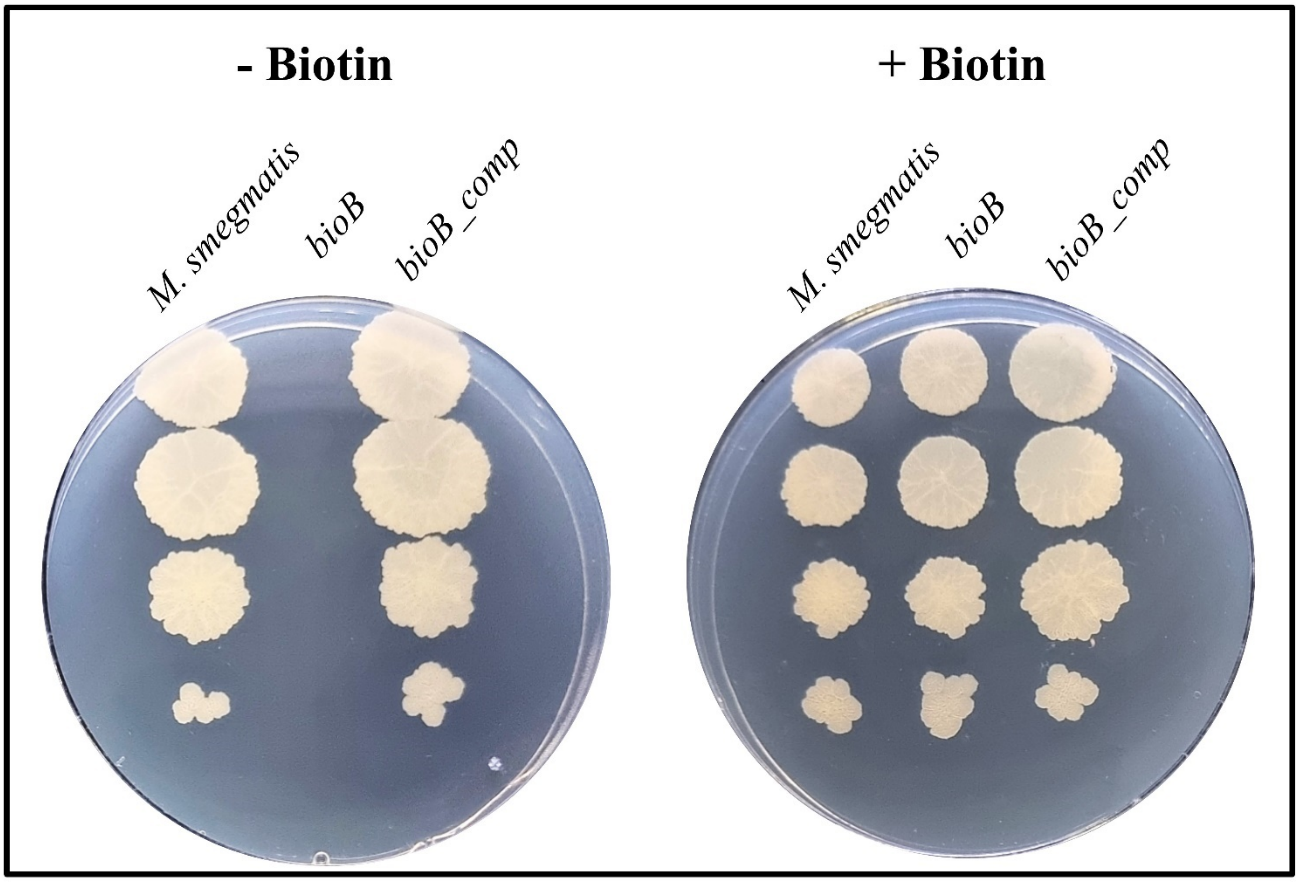


**Figure S2**. A transposon insertion in *bioB* impairs growth on a medium lacking biotin. Growth of *M. smegmatis*, *bioB* mutant, and *bioB* mutant complemented with functional *bioB* gene (*bioB_comp*) on biotin-free (left panel) or biotin-containing media (right panel). Rows are tenfold serial dilution.
